# Compared to other front-of-pack nutrition labels, the Nutri-Score emerged as the most efficient to inform Swiss consumers on the nutritional quality of food products

**DOI:** 10.1371/journal.pone.0228179

**Published:** 2020-02-27

**Authors:** Manon Egnell, Pilar Galan, Nathalie J. Farpour-Lambert, Zenobia Talati, Simone Pettigrew, Serge Hercberg, Chantal Julia

**Affiliations:** 1 Nutritional Epidemiology Research Team (EREN), Sorbonne Paris Cité Epidemiology and Statistics Research Center (CRESS), U1153 Inserm, U1125, Inra, Cnam, Paris 13 University, Bobigny, France; 2 Department of Primary Care, University Hospitals of Geneva, Geneva, Switzerland; 3 School of Psychology, Curtin University, Bentley, WA, Australia; 4 The Georges Institute, Sidney, Australia; 5 Public health department, Avicenne Hospital, AP-HP, Bobigny, France; Universitat Wien, AUSTRIA

## Abstract

**Background:**

Switzerland, like other high-income countries, is facing a major public health challenge with the increasing burden of non-communicable diseases. Discussions are currently on-going in Switzerland regarding the implementation of a Front-of-Pack nutrition label (FoPL) as a public health measure to guide consumers towards healthier food choices, and the Nutri-Score represents an alternative supported by multiple actors. To date, no studies have investigated the performance of the Nutri-Score among Swiss consumers. This study aimed to compare the response of Swiss consumers to five FoPLs (Health Star Rating system, Multiple Traffic Lights, Nutri-Score, Reference Intakes and Warning symbol) in terms of perception and understanding of these labels and effects on food choices.

**Methods:**

In 2019, 1,088 Swiss consumers were recruited and asked to select one product from among a set of three foods with different nutritional profiles and then classify the products within the sets according to their nutritional quality. Tasks were performed in situations without a label and then with one of the five FoPLs–depending on the group in which they were randomized–on the pack. Finally, participants were questioned on their perceptions regarding the label to which they were exposed.

**Results:**

All FoPLs were favorably perceived, with marginal differences between FoPLs. The Nutri-Score demonstrated the highest percentage of improvement in food choices and the highest overall performance in helping consumers rank the products according to their nutritional quality.

**Conclusion:**

Overall, the Nutri-Score was the most efficient FoPL in informing Swiss consumers of the nutritional quality of food products, and as such could be a useful tool to improve food choices and reduce the burden of chronic diseases in Switzerland.

## Introduction

As is the case in other high-income countries, Switzerland is facing a major public health challenge in the form of the increasing burden of Non-Communicable Diseases (NCDs) [1–6]. According to a report of the Swiss Federal Office of Public Health published in 2017, 80% of the direct and indirect human health costs in Switzerland were due to NCDs, notably including cancers, diabetes and cardiovascular diseases [7]. Nutritional risk factors have been recognized worldwide as some of the main drivers of these NCDs, and they therefore constitute key levers to public health policies because they represent modifiable determinants of health that could be addressed through primary prevention interventions [1–6]. According to the Nutrition Survey *MenuCH* published in 2017, Swiss people consume too much sweet, salty and meat products, and not enough legumes, fruits, vegetables and dairy products [8]. The prevalence rates of overweight and obesity are 41.6% and 13.9% in men and 19.7% and 11.3% in women [8]. In this context, the Swiss nutritional strategy for the 2017–2024 period aims to improve the nutritional status of the population and prevent NCDs by enhancing the food environment and assisting consumers to make healthier food choices [7].

Internationally, among the variety of possible interventions, Front-of-Pack nutrition Labels (FoPLs) have received growing attention from public health authorities [9–11]. They have been demonstrated to be efficient tools to help consumers make healthier food choices at the point-of-purchase as they deliver at-a-glance nutritional information [12–14]. Moreover, FoPLs act as an incentive for manufacturers to improve the nutritional quality of their products through innovation and reformulation [15,16]. In Switzerland, discussions are currently ongoing regarding the implementation of FoPLs on pre-packed foods. Public health authorities in the field of food (i.e. Swiss Federal Food Safety and Veterinary Office), consumer associations and some manufacturers support the introduction of the Nutri-Score, which is a simplified labelling system designed to reflect the overall nutritional quality of food products. The Nutri-Score is a summary and graded FoPL that can serve as a guide for consumers and help them make informed choices [17]. It uses a 5-color scale (from dark green to dark orange) with associated letters (from A to E) to indicate the overall nutritional quality of foods according to a nutrient profiling system that takes into consideration both unfavourable food composition elements for which consumption should be limited (energy, total sugars, Saturated Fatty Acids—SFA, and sodium) and favourable elements for which consumption should be encouraged (fruits, vegetables and nuts, fibre and protein). The Nutri-Score was originally developed in France and has now also been adopted in Belgium and Spain.

While studies have shown the relative superiority of the Nutri-Score compared to other label formats in various countries [18], in particular in France [17], no studies to date have investigated the performance of the Nutri-Score (and other FoPLs) among Swiss consumers. According to the theoretical framework from Grunert et *al*., defining the efficiency of FoPLs requires taking into considerations the different aspects of their validation, including notably consumer preferences/perception, understanding of the labels and their effects on declared food choices or real food purchases in real-world or naturalistic experimental trials [19]. These different dimensions (perception, understanding, use) have been suggested to be influence by FoPL format and sociodemographic and individual characteristics of consumers [19]. Studies investigating preferences suggest that most commonly used FoPLs are generally positively perceived [20,21], however favourable perceptions may not be adequate predictors of the extent to which individual FoPLs can inform consumers of the nutritional quality of products and guide their choices toward healthier foods [22]. By contrast, objective understanding, defined as the capacity for consumers to correctly interpret the information that is provided by the label as intended by its designers [19], is a superior indicator as it demonstrates the capacity of the FoPL to help consumers rank food products according to their nutritional quality. Finally, studies measuring the effects on food purchases in virtual or real supermarkets are more convincing to define the efficiency of a specific FoPL [23–33]; nevertheless experimental tasks on food choices on a limited number of products are usually performed to avoid the technical and financial constraints of studies in real-life conditions.

The objective of the present study was to inform current FoPL deliberations in Switzerland by assessing the relative effectiveness of the Nutri-Score and four other FoPLs: Multiple Traffic Lights (introduced in the United Kingdom), Health Star Rating system (implemented in New Zealand and Australia), Warning symbol (introduced in Chile) and Reference Intakes (promoted by agro-food-industries worldwide). We used the FOP-ICE study methodology that was used to compare the effectiveness of FoPLs in 12 countries [18] by investigating three dimensions: consumers’ perceptions and objective understanding of five FoPLs and their resulting food choices.

## Materials and methods

### Population study

A total of 1,088 Swiss adults were recruited through a web panel provider (Pureprofile), applying quotas for sex (50% of women), age (one third in each of the following categories: 18–30 years, 31–50 years, over 51 years) and monthly household income (one third in each of the following categories: low, medium and high). Panel members were invited to complete an online survey and could choose to do so in French, German or Italian. At the beginning of the survey, participants were asked to provide information on sex, age, monthly household income, education level, involvement in grocery shopping, self-estimated diet quality and self-estimated level of nutrition knowledge. They were also asked to declare the frequency of purchase of the tested food categories (pizzas, cakes, breakfast cereals) on a four-point scale (“Always”, “Often”, “Sometimes” and “Never”). Those who responded “Never” to at least two of the three food categories were excluded to ensure responses reflected real-world food choice behaviors. The protocol of the study (similar to the FOP-ICE study) was approved by the Institutional Review Board of the French Institute for Health and Medical Research (IRB Inserm n°17–404 bis) and the Curtin University Human Research Ethics Committee (approval reference: HRE2017-0760). Participants were invited to provide their electronic consent during the online survey.

### Front-of-pack nutrition labels

**Fig 1:**
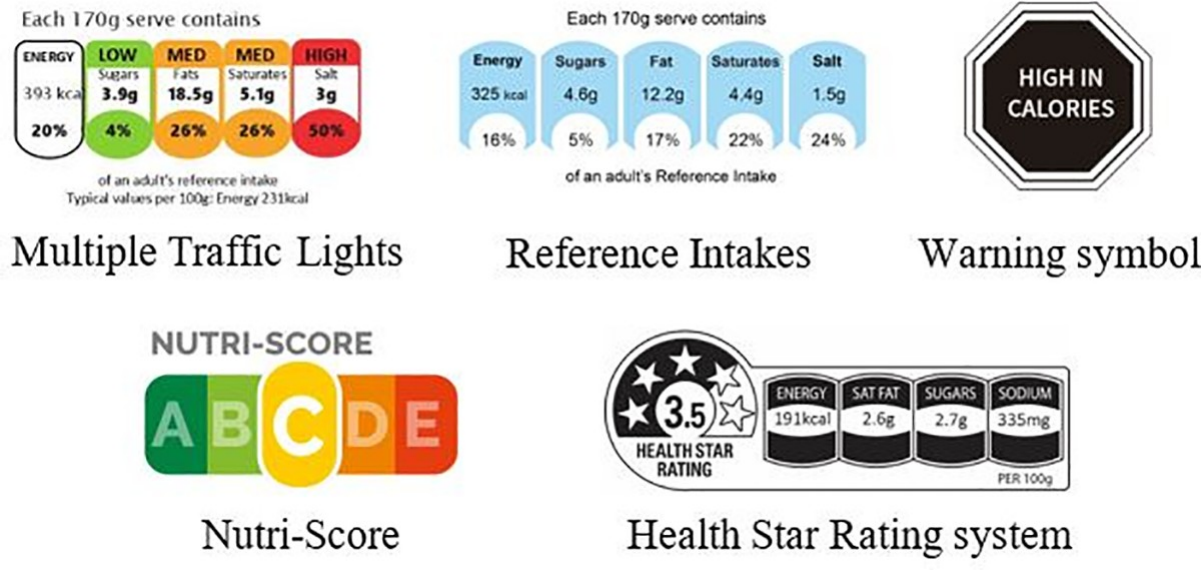
Front-of-pack nutrition labels tested in the present study. Three nutrient-specific FoPLs were included: (1) a numeric-only monochromatic label, the Reference Intakes, that was implemented worldwide in 2006 following a voluntary initiative of industrialists and displays the amounts in energy, fats, SFA, sugars and salt [35]; (2) a color-coded label, the Multiple Traffic Lights, implemented in the United Kingdom in 2004, that indicates the amounts of the same nutrients as the RIs, but with a colour associated with each nutrient depending on the amount (green—low, orange—moderate, red—high) [36]; and (3) a warning system, the Warning symbol implemented in Chile in 2016 and then in Peru in 2019, that advises when the level of a given unfavourable nutrient exceeds the limit established by the Chilean Ministry of health [37]. Second, two summary FoPLs were tested: (1) a graded color-coded label, the Nutri-Score, implemented in France in 2017 and later in 2018 in Belgium and Spain, that characterizes the overall nutritional quality of the food or beverage using a graded scale of five colors from dark green (associated with the letter A) to dark orange (associated with the letter E) [17] and (2) a hybrid FoPL, the Health Star Rating system, implemented in Australia and New Zealand in 2014, that combines a graded scale of stars and information on nutrient amounts [38].

Five FoPLs with different type of graphical designs were tested in the present study (**Fig 1** [34]).

### Design and stimuli

Three food categories (pizzas, cakes, and breakfast cereals) were tested in the present study and were selected due to being commonly available in Swiss supermarkets and incorporating products with wide variability in nutritional quality. In each food category, a set of three products with distinct nutrient profiles (higher, medium, and lower nutritional quality) was created, allowing a ranking of products according to their nutritional quality. The ranking of the relative nutritional quality between the three products was made depending on the information provided by the FoPLs, and was similar whatever the FoPL. To avoid potential bias in product evaluation (e.g., familiarity, habit), mocks packages featuring a fictional brand (“Stofer”) were developed. When FoPLs were applied to the mock packages, they were affixed in the same place on each food product and covered the same area on the package. To avoid unduly influencing participants’ perceptions of the food products, no other nutritional information or quality indicators was provided. All stimuli are displayed in **S1, S2** and **S3 Figs**.

### Procedure

**Fig 2:**
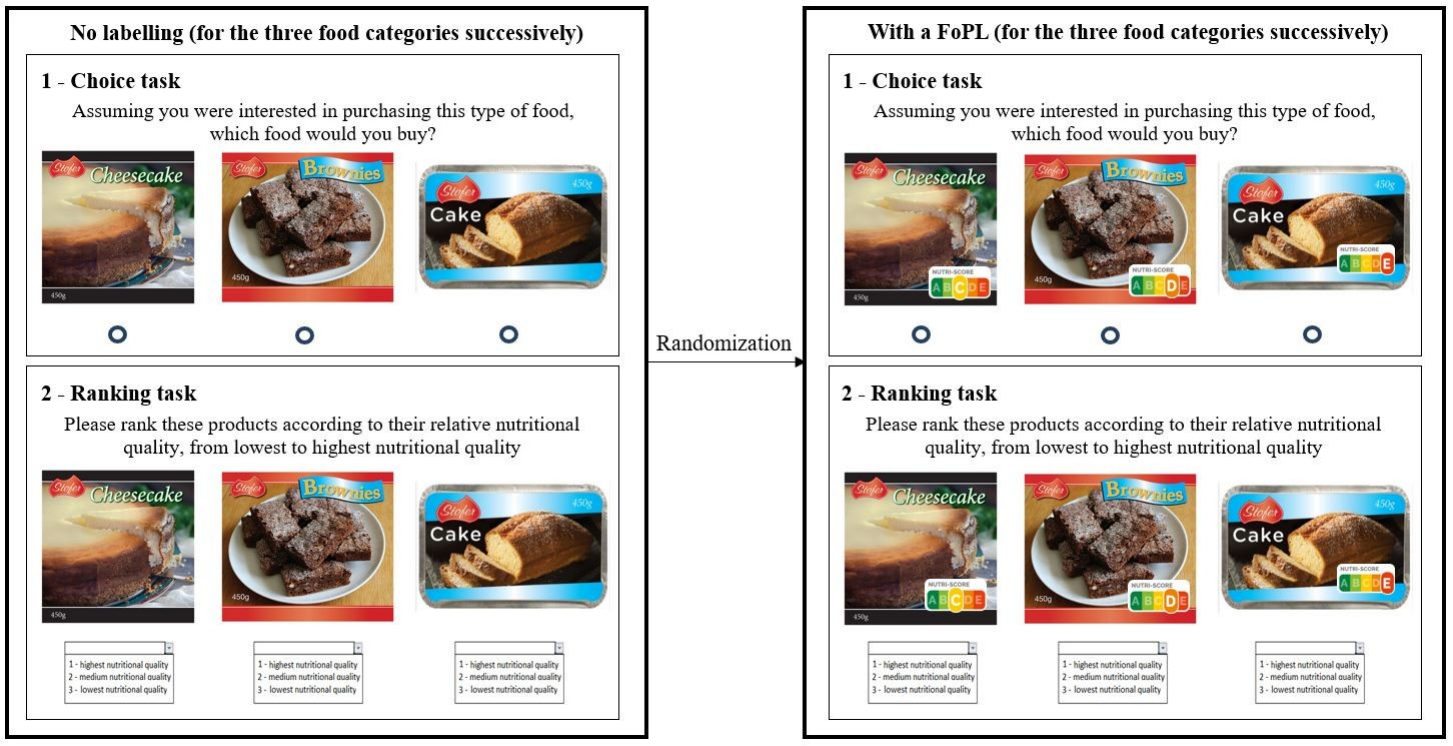
Procedure of the choice and ranking tasks for the cakes category. After the choice and ranking tasks, participants were invited to respond to questions about their perceptions on the FoPL to which they had been exposed. Various dimensions were assessed including liking (e.g. “I like this label”), usefulness (e.g. “This FoP label is useful”), awareness (e.g. “This FoP label stands out”), and perceived cognitive workload for the comparison of pre-packed foods within the same food category (e.g. “This label is easy to understand”). For each question, respondents provided their responses on a 9-point Likert scale ranging from “Strongly disagree” to “Strongly agree”.

Following the sociodemographic, lifestyle and nutrition-related questions at the beginning of the survey, participants were asked to complete choice and understanding tasks, and then to answer questions about their perceptions of the FoPL to which they had been assigned. To avoid priming participants towards paying attention specifically to the FoPLs and modify their choices accordingly by introducing first questions on perception and understanding [19], the investigation of the dimensions was performed using the reversed order: food choice, objective understanding and finally perception. First, participants were exposed to the three stimulus sets (one for each food category) without any label on the front of mock packages. They were asked to nominate which of the three displayed products they would buy, with an “I wouldn’t buy any of these products” option also available. After each choice task, participants were asked to rank the set of three products according to their nutritional quality (1- Highest nutritional quality, 2- Medium nutritional quality, and 3- Lowest nutritional quality), with an “I don’t know” option also available. The phrasing of the task used relative terms on nutritional quality (highest/medium/lowest) in order to prevent participants from making assumption on the absolute nutritional quality of the products. Choice and ranking tasks were completed by food category, successively, with the order of presentation of the food categories randomized between respondents. Second, participants were randomized to one of the five FoPL groups and asked to complete the same choice and ranking tasks, but this time with a FoPL affixed to the mock packages. An example of the procedure for the cakes category is presented in **Fig 2** [34].

### Statistical analyses

#### Food choice

A score between 1 and 3 points was attributed to the choice task of each food category, with +1 for the lowest nutritional quality product, +2 for the intermediate nutritional quality product and +3 points for the highest nutritional quality product, first for the no labelling condition and second in the FoPL condition. No point was allocated when participants selected “I wouldn’t buy any of these products” option, and the response was considered as missing. A score was then calculated for each food category using the difference of points between the FoPL and no label conditions, resulting in a discrete continuous score ranging from -2 to +2 points. Finally, a global score was computed by summing the score of each category, resulting in a score between -6 and +6 points for each participant. The percentage of participants whose food choices deteriorated or improved between the no label and FoPL conditions was calculated for each FoPL group by food category. Associations between choice score and FoPL type were assessed using a multivariable ordinal logistic regression model. The model was performed on data from participants who selected a product in both the no label and FoPL conditions.

#### Objective understanding

Objective understanding of the FoPLs by consumers was measured by the ability of participants to correctly rank the products within each set according to nutritional quality. The ranking was considered correct when the three products within the set were correctly ranked, leading to a +1 point score for the category, while -1 point was allocated when the ranking was incorrect. No point was allocated when participant selected the “I don’t know” answer. Thus, for each food category, a score for ranking accuracy was calculated using the difference in points between the FoPL and no label conditions, ranging from -2 to +2 points, and leading to a global score of between -6 and +6 points for the three food categories combined. The percentage of correct answers was computed by FoPL and food category and displayed in a histogram. The association between FoPL type and the change in ability to correctly rank products according to nutritional quality was measured by an ordinal logistic regression model.

For choice and understanding analyses, sex, age, level of household monthly income, educational level, involvement in grocery shopping, self-estimated diet quality and nutrition knowledge and the response to “In the second half of this study, the food products contained a nutrition label (example shown below). Do you remember seeing this label on products?” were introduced as covariates.

The reference of the models (for choice and understanding analyses) was the Reference Intakes label. Interactions between covariates and FoPLs were tested and stratified models were computed when the p-value of the interaction term was below 0.10.

#### Perception

For each item on perception of the FoPLs, participants provided a rating between 1 (corresponding to the statement “I strongly disagree”) and 9 (corresponding to the statement “I strongly agree). The mean and standard deviation of scores were calculated for each item and by FoPL type. A principal component analyses was performed to assess the contribution of the different perception items to the overall perception of FoPLs. The items “This label is confusing”, “I like this label”, “This label does not stand out”, “This label is easy to understand”, “This label takes too long to understand”, “This label provides me the information I need” and “I trust this label” were used as active variables in the analyses, and the label type as an illustrative qualitative variable. Dimensions, corresponding to a linear combination of active variables, have an eigenvalue reflecting the total variance explained by the dimension. The number of retained dimensions was chosen to obtain a cumulative percentage of acceptable variance. In the present analyses, only the two first dimensions were chosen, simplifying the presentation of results. The contribution and coordinates of each active variable on the two axes were obtained and the label variable was mapped on the axes as an illustrative variable. Test values were provided for the label variable, allowing testing the significance of the deviation from the origin of the qualitative variable. This difference can be considered significant at 95% level if the test value is greater than or equal to 2 in absolute value [39]. Due to the combination of positive and negative framing of the perception questions, participants who provided the same answers to all perception questions were excluded from the analyses, except those consistently giving a score of 5, which indicates a neutral perception.

All analyses in the present study were conducted on the SAS statistical software; statistical tests were two-sided and a p-value ≤ 0.05 was considered statistically significant.

## Results

### Description of individual characteristics

Sociodemographic, lifestyle and nutrition-related characteristics of the study population are presented in **Table 1**. The sample included 1,088 Swiss participants, of whom 49% were women, 35% were individuals over 51 years, 36% had a primary or secondary education level, and 32% reported a low household monthly income. In the sample, 66% declared being responsible for grocery shopping, 20% reported a very or mostly unhealthy diet quality, and 28% had no or little knowledge about nutrition. A total of 29% of participants declared that they did not recall having seen the label during the survey, with the highest percentage evident among those assigned to the Health Star Rating System group.

**Table 1:** Individual characteristics of the study sample (N = 1,088).

	N	%
**Sex**		
Men	560	51.47
Women	528	48.53
**Age, years**		
18–30	342	31.43
31–50	371	34.10
≥ 51	375	34.47
**Education level**		
Primary education	68	6.25
Secondary education	326	29.96
Trade certificate	371	34.10
University, undergraduate degree	189	17.37
University postgraduate degree	134	12.32
**Level of household monthly income**		
High	367	33.73
Medium	371	34.10
Low	350	32.17
**Responsible for grocery shopping**		
Yes	718	65.99
No	86	7.90
Share job equally	284	26.10
**Self-estimated diet quality**		
I eat a very unhealthy diet	20	1.84
I eat a mostly unhealthy diet	196	18.01
I eat a mostly healthy diet	769	70.68
I eat a very healthy diet	103	9.47
**Nutrition knowledge**		
I do not know anything about nutrition	22	2.02
I am not very knowledgeable about nutrition	288	26.47
I am somewhat knowledgeable about nutrition	579	53.22
I am very knowledgeable about nutrition	199	18.29
**Did you see the FOP label during the survey?**		
No	313	28.77
Unsure	105	9.65
Yes	670	61.58
**Respondents recalling seeing the FoPL to which they were exposed**		
*HSR*	122	55.96
*MTL*	145	66.82
*Nutri-Score*	164	75.23
*RIs*	143	65.90
*Warning symbol*	196	89.91

HSR: Health Star Rating system; MTL: Multiple Traffic Lights; RIs: Reference Intakes

### Food choices

**Fig 3:**
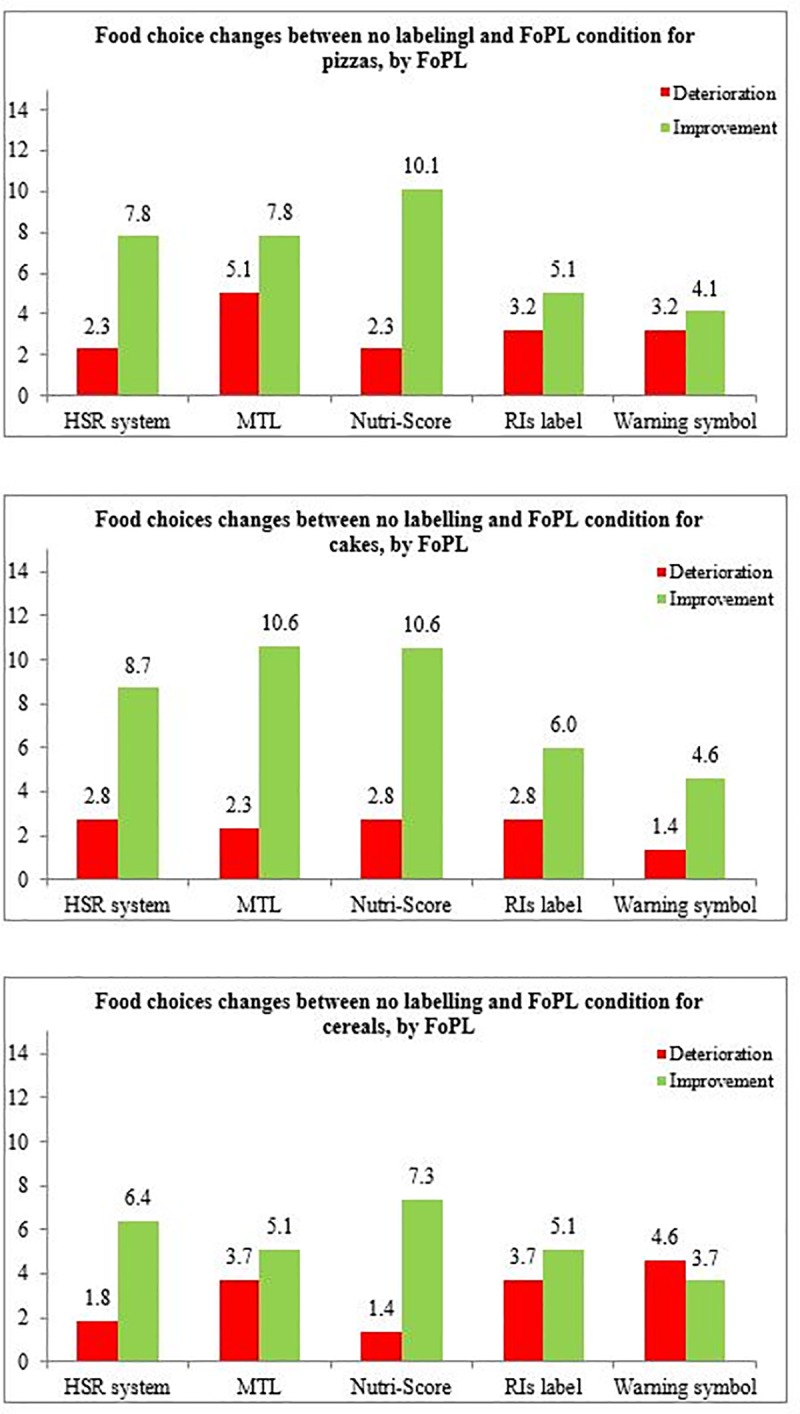
Percentages of deterioration and improvement of the nutritional quality of food choices, by FoPL type and food category. Associations between FoPL type and food choices are displayed in **Table 2**. The Nutri-Score was the only FoPL to demonstrate a significant effect on the improvement of the nutritional quality of food choices compared to the RIs label. This occurred overall (OR = 1.83[1.17–2.86], p-value = 0.008) and among pizzas (OR = 1.90[1.01–3.57], p-value = 0.05).

Most of the participants did not change their food choices between the two labelling situations (between 58.1% and 71.0% depending on the label and the food category) or did not select any product in one or both of the labelling conditions (between 20.7% and 35.3%, depending on the label type and the food category). The percentages of participants who improved or deteriorated in their choices between the FoPL and no label conditions are shown in **Fig 3**. For all three food categories and all five FoPLs, the percentage of participants who improved their food choices between the two labelling conditions was higher than those whose choices deteriorated, however results varied depending on the label. The Nutri-Score demonstrated the greatest improvement (between 7.3% and 10.6% depending on the food category), while the RIs (3.7% - 4.6%) and the Warning symbol (5.1% - 6.0%) showed the smallest improvement.

**Table 2:** Associations between FoPL type and change in nutritional quality of food choices, by FoPL type and food category in participants who made a choice ^a^ (N = 1,000).

Food category	N	HSR		MTL		Nutri-Score		Warning symbol	
		OR (95% CI)	P	OR (95% CI)	P	OR (95% CI)	P	OR (95% CI)	P
All food categories	1000	1.44 [0.91–2.28]	0.1	1.18 [0.74–1.88]	0.5	1.83 [1.17–2.86]	0.008	0.89 [0.56–1.44]	0.6
Pizzas	834	1.56 [0.82–2.96]	0.2	1.14 [0.60–2.19]	0.7	1.90 [1.01–3.57]	0.05	1.09 [0.56–2.12]	0.8
Cakes	781	1.41 [0.74–2.69]	0.3	1.74 [0.92–3.27]	0.09	1.62 [0.86–3.03]	0.1	1.26 [0.64–2.50]	0.5
Breakfast cereals	779	1.49 [0.74–3.02]	0.3	0.94 [0.46–1.90]	0.9	1.57 [0.79–3.12]	0.2	0.75 [0.36–1.54]	0.4

^a^ The Reference Intakes were designated as the reference category for the ‘labels’ variable in the multivariate ordinal logistic regression.

The multivariate model was adjusted for sex, age, education level, level of income, responsibility for grocery shopping, self-estimated diet quality, self-estimated nutrition knowledge and awareness of the label during survey completion

HSR: Health Star Rating system; MTL: Multiple Traffic Lights; OR: Odds Ratio; CI: Confidence Interval.

A significant interaction was observed with household monthly income (**S1 Table**). While all labels tended to have a greater effect on food choices than the RIs among those on medium incomes, the MTL and the Warning symbol were significantly less effective than the RIs among individuals on low incomes.

### Objective understanding

**Fig 4:**
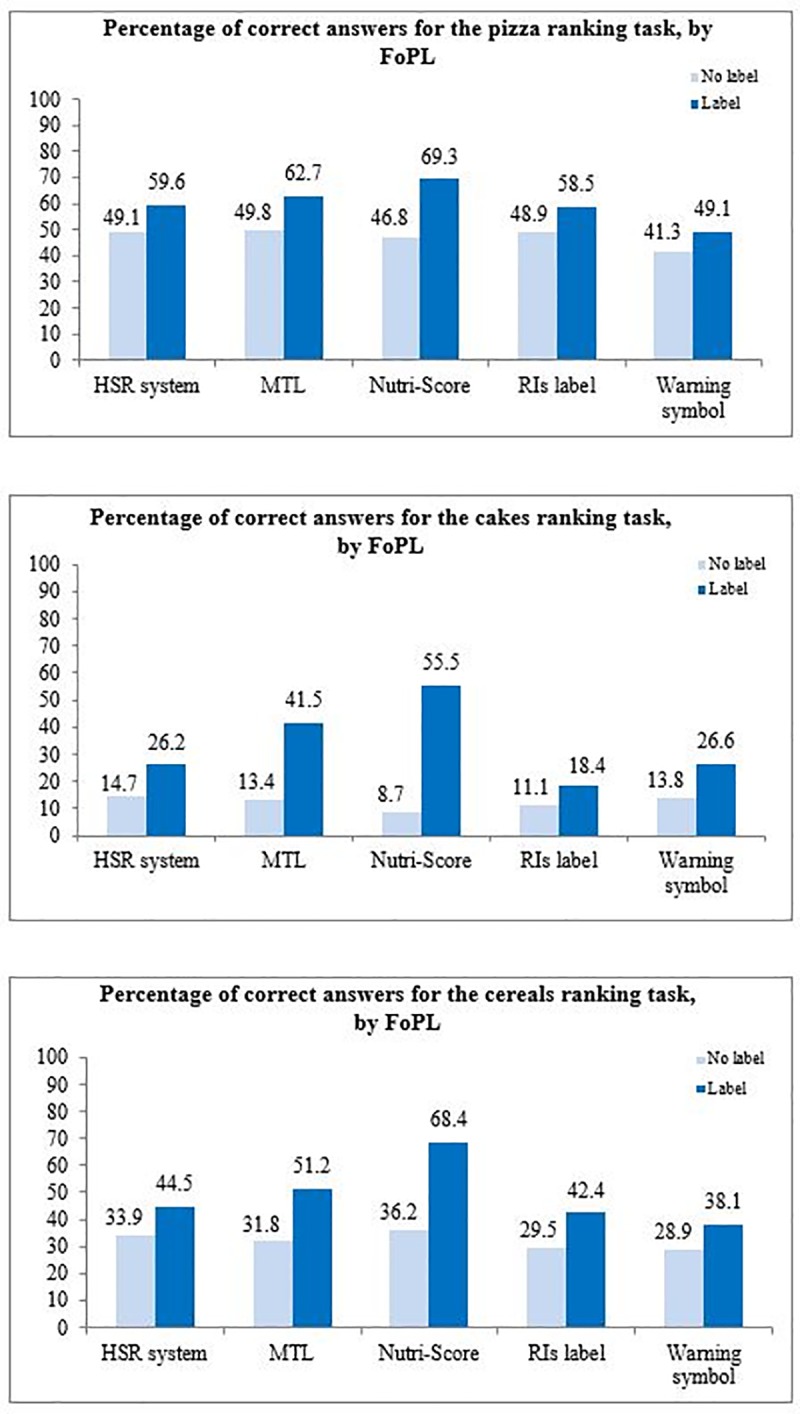
Percentage of correct answers for ranking tasks, by FoPL and food category. Associations between FoPL type and ability to correctly rank products are presented in **Table 3**. Overall, the Nutri-Score was the label leading to the greatest improvement in ability to correctly rank products according to their nutritional quality compared to the RIs (OR = 4.02[2.81–5.75] (p-value<0.0001), followed by the MTL (OR = 2.09[1.46–2.99], p-value<0.0001) and the Warning symbol (OR = 1.52[1.05–2.18], p-value = 0.03). When analyses were performed by food category, the Nutri-Score showed higher performances among the three categories, and was notably the only FoPL to show significant improvements compared to the RIs label among pizzas and breakfast cereals. Among cakes, the performance of the Nutri-Score was followed by the MTL, the Warning symbol and then the HSR.

The percentages of correct answers in the no label and label conditions by FoPL type and food category are shown in **Fig 4**. Compared to the no label condition, all FoPLs improved the percentage of correct answers, with some heterogeneous results between labels formats. For all three food categories, the Nutri-Score produced the largest improvement in correct answers in the ranking tasks, followed by the MTL. The relative performance of the other FoPLs varied by food category.

**Table 3:** Associations between FoPLs and the ability to correctly rank products according to nutritional quality, by FoPL and food category ^a^ (N = 1,088).

Food category	N	HSR		MTL		Nutri-Score		Warning symbol	
		OR (95% CI)	P	OR (95% CI)	P	OR (95% CI)	P	OR (95% CI)	P
All categories	1088	1.43 [1.00–2.05]	0.05	2.09 [1.46–2.99]	<0.0001	4.02 [2.81–5.75]	<0.0001	1.52 [1.05–2.18]	0.03
Pizzas	1034	1.43 [0.89–2.30]	0.1	1.50 [0.94–2.40]	0.09	2.36 [1.49–3.72]	0.0002	1.39 [0.86–2.26]	0.2
Cakes	1039	1.64 [1.06–2.54]	0.03	3.11 [2.03–4.78]	<0.0001	5.97 [3.90–9.15]	<0.0001	2.09 [1.35–3.25]	0.001
Breakfast cereals	1006	1.05 [0.68–1.64]	0.8	1.29 [0.83–1.98]	0.3	2.25 [1.47–3.43]	0.0002	1.03 [0.65–1.61]	0.9

^a^ The Reference Intakes were designated as the reference category for the ‘labels’ variable in the multivariate ordinal logistic regression.

The multivariate model was adjusted for sex, age, educational level, level of income, responsibility for grocery shopping, self-estimated diet quality, self-estimated nutrition knowledge level and awareness of the label during survey completion.

HSR: Health Star Rating system; MTL: Multiple Traffic Lights; OR: Odds Ratio; CI: Confidence Interval.

No interaction with individual characteristics was found, except for age and self-estimated diet quality. However, the interactions were quantitative, meaning that FoPLs improved the participants’ ability to correctly rank products among all variable categories (**S2 and S3 Tables**).

### Perception

All results on FoPLs perception are presented in supporting information. The average scores for all perception questions are displayed in **S4 Fig**. Overall, similar trends were found for the five FoPLs on the different perception items.

The principal component analysis identified two main dimensions explaining 45.9% and 17.8% of the total variance respectively. The contribution values and coordinates of active variables on these two dimensions are displayed in **S4 Table**. The first dimension (horizontal axis) opposed the items “I like this label”, “This label is easy to understand” and “This label provides me the information I need” with the items “This label is confusing” and “This label takes too long to understand”. The second dimension (vertical axis) was driven by the item “This label does not stand out”.

When each label was mapped on the two axes as an illustrative variable, the graphic in **S5 Fig** was obtained. Although differences between FoPLs on the two dimensions appeared of very low magnitude, the MTL appeared to be perceived as providing the “information needed”, “being easy to understand” and “likeable”. Regarding the second dimension, the Nutri-Score was perceived as “standing out” to a greater extent than the RIs and the Warning symbol, both monochromatic formats (test values greater than 2 in absolute value).

## Discussion

Overall, among the various FoPLs tested in the study, our results showed that the Nutri-Score was the most effective scheme in encouraging healthier food choices among study participants and allowing them to more accurately identify differences in the nutritional quality of foods within product categories.

Many studies have explored the effects of different types of FoPLs on the nutritional quality of consumers’ food choices or purchases, with mixed results according to the types of FoPLs tested and/or the methodology used [21,23,28,29,31–33,40–69]. These studies suggest that FoPLs can induce a small but significant beneficial effect on the nutritional quality of food choices/purchases. Interpretive systems in particular, such as Nutri-Score [29,31,32], Multiple Traffic Lights [29,33,45,48,55,65], Health Star Rating [31,46] and warning labels [28,41,42,54] appear to be associated with healthier food choices. Moreover, comparative studies investigating the relative effects of various types of labels indicate limited differences between types of FoPLs regarding their effects on food choices [26,27,29]. Our results regarding the Nutri-Score’s effect on food choices are consistent with those of other studies investigating the impact of the Nutri-Score in purchasing situations in France: experimental studies asking participants to perform a shopping task in the presence or absence of a FoPL showed that, among several schemes, the Nutri-Score was the most effective in improving the nutritional quality of purchases [29–31]. This alignment of results in neighboring countries may be related to similar socio-cultural contexts and similar food culture. By comparison, results from the Americas (Canada, Uruguay) suggest warning labels would be more effective among consumers from these countries [26,28]. However, given the varied methodological approaches used in the different published studies to investigate the effects of FoPLs on food choices, caution is required before concluding on this unique basis on the effectiveness of a given type of label. Robustness of proof is higher when testing the impact of different FoPL on real food purchases in real-world or naturalistic experimental trials. However, given the somewhat low magnitude of effects observed, conducting adequately powered studies would require high resources. In this case, our results suggest that if studies testing FoPL on food purchases in virtual or real supermarkets are not available, performance would be best approached by investigating the relative ability of different FoPLs to help consumers understand the nutritional quality of foods (i.e. through measures of objective understanding). Indeed, the effects of FoPLs on consumers’ ability to correctly rank products according to their nutritional quality were of higher magnitude than their effects on food choices (ORs ranging from 1.52 for the warning symbol to 4.02 for the Nutri-Score for objective understanding vs. 0.89 for the warning symbol to 1.82 for the Nutri-Score for choice).

Second, the results for objective understanding allow to discriminate across FoPLs, with the Nutri-Score having a higher performance than other labels. These findings are in line with the results of the FOP-ICE study and subsequent studies using the same methodology that showed that the Nutri-Score had a significantly greater ability to help consumers rank the overall nutritional quality of food products in numerous European countries: France, Germany, Spain, the United Kingdom, Denmark, and Bulgaria [18,34,70]. Results in the Netherlands using the same methodology of the FOP-ICE study showed also similar trends [34]. The literature shows that labels including some form of color-coding are easier to identify and interpret [71,72], and red, green and yellow/amber on food packages are directly associated with evaluation of products’ healthfulness by consumers [73], and interpreted as ‘stop’ and ‘go’ signals [74]. This element is somewhat strengthened by the fact that the HSR, which uses a similar algorithm to classify foods, and provides a monochrome translation of the information had a lower performance than Nutri-Score. Conversely, nutrient-specific systems, and in particular those relying heavily on numerical information, require a cognitive workload that can hinder their understanding and use in purchasing situations. These elements suggest that the key features of the Nutri-Score that may in part explain its performance are the use of color-coding and of a summary indicator of the nutritional quality of the product [18,71,75]. However, the use of such simplified messaging may be associated to halo effects in products favourably labeled, which should be further investigated in the specific case of FoPLs. Effects of a FoPL on consumers’ objective understanding of the nutritional quality of foods and on their food choices provide an evaluation of the performance of the system, linked to its potential impact on the nutritional and health status of the population [76]. The fact that the effects of the Nutri-Score aligned on these two dimensions in this study suggest it would indeed be an effective intervention for the Swiss population.

Finally, consumers’ perceptions of FoPLs suggest that all five types of labels tested in the present study are considered acceptable by consumers, with limited discrimination across schemes. As respondents only viewed one FoPL, our results may be interpreted as indicating an overall favorable perception of FoPLs in the sample rather than an absence of preference towards a specific scheme [77]. Indeed, consumers tend to agree on the fact that the back-of-pack nutritional declaration is difficult to understand [78,79], and the demand for simplified front-of-pack labels [13] is increasing as evidenced by the current upward trend in implementation of FoPLs around the world [80]. Results from studies presenting various FoPL models to consumers suggest that color-coded labels would be preferred by consumers [50,72,81], and summary systems more specifically by more disadvantaged groups [82].

Strengths of our study include the use of a randomized design to compare the effects of various types of FoPL designs across their three main dimensions (effect on choice, ability to improve assessment of nutritional quality, and consumer perceptions). As randomization was applied to the order of presentation of the food categories and the order of presentation of the foods within the sets, a potential learning effect was avoided. Our study is nevertheless subject to limitations. First, Swiss consumers were recruited online using quota sampling, and as such caution is required when extrapolating the results to the broader population. However, the quota sampling ensured that various socio-economic groups were equally represented in our sample, particularly lower income groups who may be a specific target for nutrition interventions. Second, to reduce priming effects, participants were blinded to the objective of the study and were provided no information on the objective or the meaning of the FoPL to which they were exposed. Participants may therefore have overlooked the information provided by FoPLs, leading to an underestimation of the labels’ effects, although it could be closer to real life conditions. Nevertheless, all FoPLs were equally impacted by this effect. Moreover, the limited information provided to participants reinforce the ecological validity of our results, given that the implementation of FoPLs in real-life settings would not necessarily be associated with extensive information provision.

In conclusion, among the different options tested in the study, the Nutri-Score appears to be the most effective FoPL to inform Swiss consumers of the nutritional quality of food products and could therefore be a helpful tool to guide consumers to integrate a nutritional dimension in purchasing situations. This point is particularly important considering that the Nutri-Score has also been shown recently in a simulation study to have the potential to decrease mortality from diet-related NCDs [76].

## Supporting information

S1 Table Associations between FoPL type and change in nutritional quality of food choices, by monthly income level, across the three food categories. (DOCX)

S2 Table Associations between FoPLs and the ability to correctly rank products according to nutritional quality, by FoPL and food category. (DOCX)

S3 Table Associations between FoPLs and the ability to correctly rank products according to nutritional quality, by FoPL and food category. (DOCX)

S4 Table Contributions and coordinates of active variables on the two dimensions from the principal component analyses. (DOCX)

S1 Fig Stimuli for the category of cakes with the corresponding front-of-pack nutrition labels. (PDF)

S2 Fig Stimuli for the category of breakfast cereals with the corresponding front-of-pack nutrition labels. (PDF)

S3 Fig Stimuli for the category of pizzas with the corresponding front-of-pack nutrition labels. (PDF)

S4 Fig Average scores for perception questions. (PDF)

S5 Fig Principal component analysis map showing projection of the FoPLs across two dimensions. (PDF)
