# Supplementary material for: Compared to other front-of-pack nutrition labels, the Nutri-Score emerged as the most efficient to inform Swiss consumers on the nutritional quality of food products
Source: PLoS One. 2020 Feb 27;15(2):e0228179. doi: 10.1371/journal.pone.0228179 (PMC7046267; doi:10.1371/journal.pone.0228179)
Supplement: S1 Table — (DOCX) [file pone.0228179.s001.docx]

**S1 Table. Associations between FoPL type and change in nutritional quality of food choices, by monthly income level, across the three food categories^a^**

| **Monthly income level** | **N** | **HSR** | | **MTL** | | **Nutri-Score** | | **Warning symbol** | |
| --- | --- | --- | --- | --- | --- | --- | --- | --- | --- |
|  |  | **OR (95% CI)** | **P** | **OR (95% CI)** | **P** | **OR (95% CI)** | **P** | **OR (95% CI)** | **P** |
| Low | 345 | 0.76 [0.33-1.74] | 0.5 | 0.42 [0.18-0.99] | 0.05 | 1.29 [0.59-2.81] | 0.5 | 0.41 [0.17-0.96] | 0.04 |
| Medium | 371 | 2.24 [0.99-5.06] | 0.05 | 2.95 [1.33-6.50] | 0.008 | 3.02 [1.35-6.75] | 0.007 | 1.10 [0.46-2.64] | 0.8 |
| High | 303 | 1.59 [0.70-3.58] | 0.3 | 0.92 [0.39-2.17] | 0.9 | 1.29 [0.57-2.88] | 0.5 | 1.22 [0.53-2.80] | 0.6 |

^a^ The Reference Intakes were designated as the reference category for the ‘labels’ variable in the multivariate ordinal logistic regressions.

The multivariate models were performed within the monthly income levels and adjusted for sex, age, education level, responsibility for grocery shopping, self-estimated diet quality, self-estimated nutrition knowledge and awareness of the label during survey completion

HSR: Health Star Rating system; MTL: Multiple Traffic Lights; OR: Odds Ratio; CI: Confidence Interval.
