# Supplementary material for: Compared to other front-of-pack nutrition labels, the Nutri-Score emerged as the most efficient to inform Swiss consumers on the nutritional quality of food products
Source: PLoS One. 2020 Feb 27;15(2):e0228179. doi: 10.1371/journal.pone.0228179 (PMC7046267; doi:10.1371/journal.pone.0228179)
Supplement: S2 Table — (DOCX) [file pone.0228179.s002.docx]

**S2 Table. Associations between FoPLs and the ability to correctly rank products according to nutritional quality, by FoPL and food category^a^**

| **Category of age** | **N** | **HSR** | | **MTL** | | **Nutri-Score** | | **Warning symbol** | |
| --- | --- | --- | --- | --- | --- | --- | --- | --- | --- |
|  |  | **OR (95% CI)** | **P** | **OR (95% CI)** | **P** | **OR (95% CI)** | **P** | **OR (95% CI)** | **P** |
| 18-30 | 342 | 2.46 [1.27-4.77] | 0.008 | 2.55 [1.29-5.04] | 0.007 | 3.56 [1.82-6.97] | 0.0002 | 1.36 [0.67-2.73] | 0.4 |
| 31-50 | 371 | 1.58 [0.85-2.93] | 0.1 | 2.35 [1.29-4.29] | 0.005 | 5.83 [3.13-10.88] | <0.0001 | 2.28 [1.22-4.29] | 0.01 |
| >50 | 375 | 0.79 [0.42-1.50] | 0.5 | 1.60 [0.86-2.95] | 0.1 | 3.63 [1.95-6.76] | <0.0001 | 1.21 [0.65-2.24] | 0.5 |

^a^ The Reference Intakes were designated as the reference category for the ‘labels’ variable in the multivariate ordinal logistic regressions.
The multivariate models were performed within age categories and adjusted for sex, educational level, level of income, responsibility for grocery shopping, self-estimated diet quality, self-estimated nutrition knowledge level and awareness of the label during survey completion.

HSR: Health Star Rating system; MTL: Multiple Traffic Lights; OR: Odds Ratio; CI: Confidence Interval.
