# Supplementary material for: Compared to other front-of-pack nutrition labels, the Nutri-Score emerged as the most efficient to inform Swiss consumers on the nutritional quality of food products
Source: PLoS One. 2020 Feb 27;15(2):e0228179. doi: 10.1371/journal.pone.0228179 (PMC7046267; doi:10.1371/journal.pone.0228179)
Supplement: S3 Table — (DOCX) [file pone.0228179.s003.docx]

**S3 Table. Associations between FoPLs and the ability to correctly rank products according to nutritional quality, by FoPL and food category^a^**

| **Self estimated diet quality** | **N** | **HSR** | | **MTL** | | **Nutri-Score** | | **Warning symbol** | |
| --- | --- | --- | --- | --- | --- | --- | --- | --- | --- |
|  |  | **OR (95% CI)** | **P** | **OR (95% CI)** | **P** | **OR (95% CI)** | **P** | **OR (95% CI)** | **P** |
| Unhealthy or very unhealthy diet | 216 | 1.62 [0.70-3.74] | 0.3 | 3.37 [1.41-8.03] | 0.006 | 9.77 [4.25-22.47] | <0.0001 | 1.11 [0.45-2.77] | 0.8 |
| Healthy or very healthy diet | 872 | 1.45 [0.97-2.18] | 0.07 | 1.90 [1.27-2.83] | 0.002 | 3.31 [2.21-4.96] | <0.0001 | 1.61 [1.08-2.41] | 0.02 |

^a^ The Reference Intakes were designated as the reference category for the ‘labels’ variable in the multivariate ordinal logistic regressions.
The multivariate models were performed within self-estimated diet quality levels and adjusted for sex, age, educational level, level of income, responsibility for grocery shopping, self-estimated nutrition knowledge level and awareness of the label during survey completion.

HSR: Health Star Rating system; MTL: Multiple Traffic Lights; OR: Odds Ratio; CI: Confidence Interval.
