# Supplementary material for: Compared to other front-of-pack nutrition labels, the Nutri-Score emerged as the most efficient to inform Swiss consumers on the nutritional quality of food products
Source: PLoS One. 2020 Feb 27;15(2):e0228179. doi: 10.1371/journal.pone.0228179 (PMC7046267; doi:10.1371/journal.pone.0228179)
Supplement: S4 Table — (DOCX) [file pone.0228179.s004.docx]

**S4 Table. Contributions and coordinates of active variables on the two dimensions from the principal component analyses**

| **Questions** | **Contributions** | | **Coordinates** | | **Test value** | | |  |
| --- | --- | --- | --- | --- | --- | --- | --- | --- |
|  | **Dimension 1** | **Dimension 2** | **Dimension 1** | **Dimension 2** | | **Dimension 1** | **Dimension 2** | |
| This label is confusing | 17.62 | 8.17 | -1.69 | 0.72 | | - | - | |
| I like this label | 16.98 | 7.18 | 1.66 | 0.67 | | - | - | |
| This label does not stand out | 4.87 | 48.16 | -0.89 | 1.74 | | - | - | |
| This label is easy to understand | 16.10 | 0.70 | 1.62 | 0.21 | | - | - | |
| This label takes too long to understand | 15.07 | 11.97 | -1.57 | 0.87 | | - | - | |
| This label provides me the information I need | 16.44 | 11.24 | 1.64 | 0.84 | | - | - | |
| I trust this label | 12.92 | 12.59 | 1.45 | 0.89 | | - | - | |
| HSR | - | - | -0.15 | -0.13 | | -0.62 | -0.84 | |
| MTL | - | - | 0.61 | -0.05 | | 2.47 | -0.30 | |
| Nutri-Score | - | - | 0.29 | -0.65 | | 1.18 | -4.24 | |
| RIs label | - | - | -0.37 | 0.44 | | -1.49 | 2.87 | |
| Warning symbol | - | - | -0.38 | 0.38 | | -1.55 | 2.52 | |

Labels do not have contribution values given that they were considered as qualitative supplementary variables and were thus not used to compute the dimensions.
