# Supplementary material for: Compared to other front-of-pack nutrition labels, the Nutri-Score emerged as the most efficient to inform Swiss consumers on the nutritional quality of food products
Source: PLoS One. 2020 Feb 27;15(2):e0228179. doi: 10.1371/journal.pone.0228179 (PMC7046267; doi:10.1371/journal.pone.0228179)
Supplement: S1 Fig — (PDF) [file pone.0228179.s005.pdf]

<table>
<tr><th>Labelling condition</th><th>Cake n°1: higher nutritional quality</th><th>Cake n°2: Medium nutritional quality</th><th>Cake n°3: Lower nutritional quality</th></tr>
<tr><td>No label</td><td>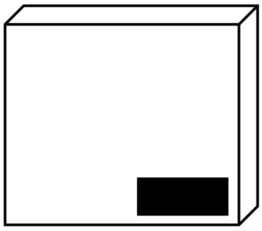</td><td>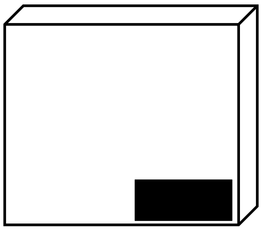</td><td>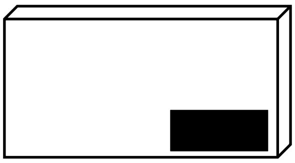</td></tr>
<tr><td>Health Star Rating system</td><td>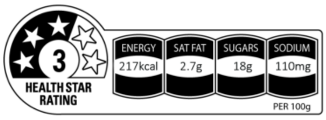</td><td>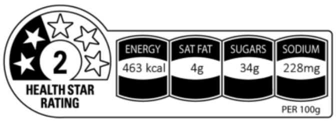</td><td>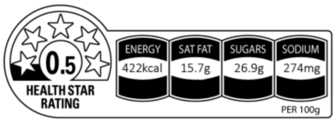</td></tr>
<tr><td>Multiple Traffic Lights</td><td>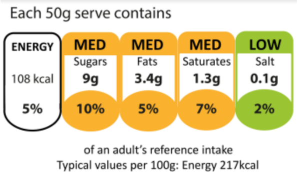</td><td>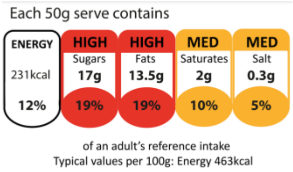</td><td>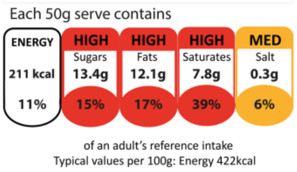</td></tr>
<tr><td>Nutri-Score</td><td>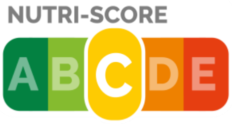</td><td>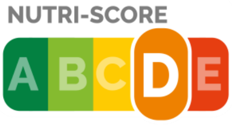</td><td>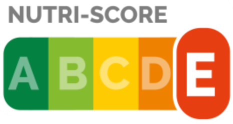</td></tr>
<tr><td>Reference Intakes label</td><td>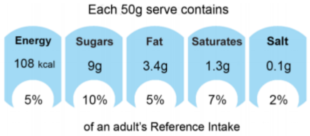</td><td>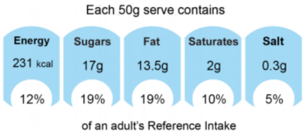</td><td>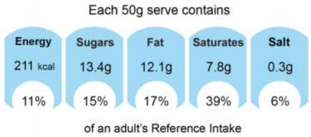</td></tr>
<tr><td>Warning symbol</td><td>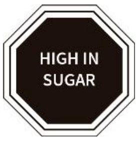</td><td>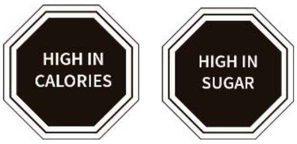</td><td>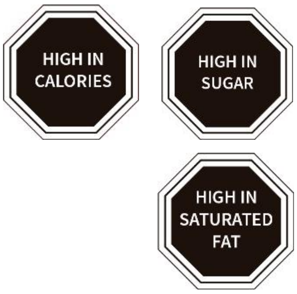</td></tr>
</table>
