# Supplementary material for: Compared to other front-of-pack nutrition labels, the Nutri-Score emerged as the most efficient to inform Swiss consumers on the nutritional quality of food products
Source: PLoS One. 2020 Feb 27;15(2):e0228179. doi: 10.1371/journal.pone.0228179 (PMC7046267; doi:10.1371/journal.pone.0228179)
Supplement: S2 Fig — (PDF) [file pone.0228179.s006.pdf]

Labelling condition	Cereal n°1: Higher nutritional quality	Cereal n°2: Medium nutritional quality	Cereal n°3: Lower nutritional quality
No label	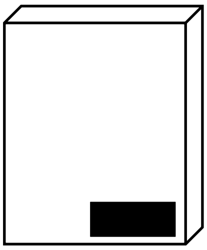	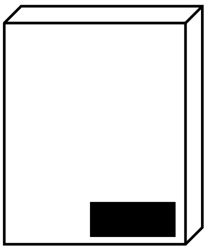	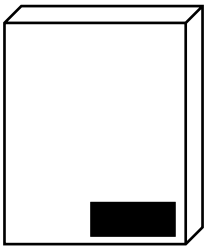
Health Star Rating system	3.5 HEALTH STAR RATING<br>ENERGY 378kcal · SAT FAT <1g · SUGARS 8g · SODIUM 390mg<br>PER 100g	2.5 HEALTH STAR RATING<br>ENERGY 387kcal · SAT FAT 1g · SUGARS 30g · SODIUM 295mg<br>PER 100g	1.5 HEALTH STAR RATING<br>ENERGY 452kcal · SAT FAT 4g · SUGARS 29g · SODIUM 445mg<br>PER 100g
Multiple Traffic Lights	Each 30g serve contains<br>ENERGY 113 kcal 6%<br>MED Sugars 2.4g 3%<br>LOW Fats 0.2g <1%<br>LOW Saturates <0.1g <1%<br>MED Salt 0.3g 5%<br>of an adult's reference intake<br>Typical values per 100g: Energy 378kcal	Each 30g serve contains<br>ENERGY 116 kcal 6%<br>HIGH Sugars 9g 10%<br>LOW Fats 0.7g 1%<br>LOW Saturates 0.3g 2%<br>MED Salt 0.2g 4%<br>of an adult's reference intake<br>Typical values per 100g: Energy 387kcal	Each 30g serve contains<br>ENERGY 136 kcal 7%<br>HIGH Sugars 8.7g 10%<br>MED Fats 4.8g 7%<br>MED Saturates 1.2g 6%<br>MED Salt 0.3g 6%<br>of an adult's reference intake<br>Typical values per 100g: Energy 451kcal
Nutri-Score	NUTRI-SCORE A **B** C D E	NUTRI-SCORE A B **C** D E	NUTRI-SCORE A B C **D** E
Reference Intakes label	Each 30g serve contains<br>Energy 113 kcal 6%<br>Sugars 2.4g 3%<br>Fat 0.2g <1%<br>Saturates <0.1g <1%<br>Salt 0.3g 5%<br>of an adult's Reference Intake	Each 30g serve contains<br>Energy 116 kcal 6%<br>Sugars 9g 10%<br>Fat 0.7g 1%<br>Saturates 0.3g 2%<br>Salt 0.2g 4%<br>of an adult's Reference Intake	Each 30g serve contains<br>Energy 136 kcal 7%<br>Sugars 8.7g 10%<br>Fat 4.8g 7%<br>Saturates 1.2g 6%<br>Salt 0.3g 6%<br>of an adult's Reference Intake
Warning symbol	HIGH IN CALORIES	HIGH IN CALORIES<br>HIGH IN SUGAR	HIGH IN CALORIES<br>HIGH IN SUGAR<br>HIGH IN SODIUM
