# Supplementary material for: Compared to other front-of-pack nutrition labels, the Nutri-Score emerged as the most efficient to inform Swiss consumers on the nutritional quality of food products
Source: PLoS One. 2020 Feb 27;15(2):e0228179. doi: 10.1371/journal.pone.0228179 (PMC7046267; doi:10.1371/journal.pone.0228179)
Supplement: S3 Fig — (PDF) [file pone.0228179.s007.pdf]

Labelling condition	Pizza n°1: higher nutritional quality	Pizza n°2: Medium nutritional quality	Pizza n°3: Lower nutritional quality
No label	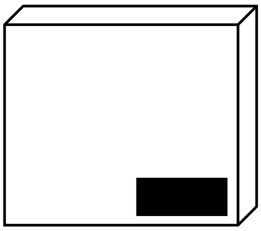	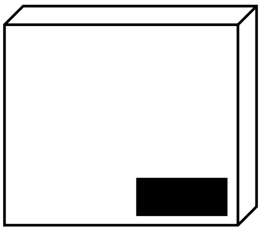	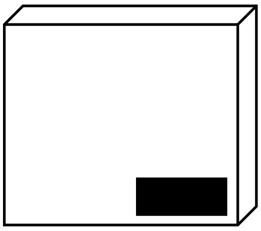
Health Star Rating System	3.5 HEALTH STAR RATING<br>ENERGY 191kcal · SAT FAT 2.6g · SUGARS 2.7g · SODIUM 335mg<br>PER 100g	2.5 HEALTH STAR RATING<br>ENERGY 231kcal · SAT FAT 3g · SUGARS 2.3g · SODIUM 689mg<br>PER 100g	1.5 HEALTH STAR RATING<br>ENERGY 274kcal · SAT FAT 8.7g · SUGARS 1.4g · SODIUM 594mg<br>PER 100g
Multiple Traffic Lights	Each 170g serve contains<br>ENERGY 325 kcal 16% · LOW Sugars 4.6g 5% · MED Fats 12.2g 17% · MED Saturates 4.4g 22% · MED Salt 1.5g 24%<br>of an adult's reference intake<br>Typical values per 100g: Energy 191kcal	Each 170g serve contains<br>ENERGY 393 kcal 20% · LOW Sugars 3.9g 4% · MED Fats 18.5g 26% · MED Saturates 5.1g 26% · HIGH Salt 3g 50%<br>of an adult's reference intake<br>Typical values per 100g: Energy 231kcal	Each 170g serve contains<br>ENERGY 466 kcal 23% · LOW Sugars 2.3g 3% · HIGH Fats 28.4g 41% · HIGH Saturates 14.7g 74% · HIGH Salt 2.6g 43%<br>of an adult's reference intake<br>Typical values per 100g: Energy 274kcal
Nutri-Score	NUTRI-SCORE A **B** C D E	NUTRI-SCORE A B **C** D E	NUTRI-SCORE A B C **D** E
Reference Intakes label	Each 170g serve contains<br>Energy 325 kcal 16% · Sugars 4.6g 5% · Fat 12.2g 17% · Saturates 4.4g 22% · Salt 1.5g 24%<br>of an adult's Reference Intake	Each 170g serve contains<br>Energy 393 kcal 20% · Sugars 3.9g 4% · Fat 18.5g 26% · Saturates 5.1g 26% · Salt 3g 50%<br>of an adult's Reference Intake	Each 170g serve contains<br>Energy 466 kcal 23% · Sugars 2.3g 3% · Fat 28.4g 41% · Saturates 14.7g 74% · Salt 2.6g 43%<br>of an adult's Reference Intake
Warning symbol		HIGH IN SODIUM	HIGH IN SODIUM · HIGH IN SATURATED FAT
