# Supplementary material for: Compared to other front-of-pack nutrition labels, the Nutri-Score emerged as the most efficient to inform Swiss consumers on the nutritional quality of food products
Source: PLoS One. 2020 Feb 27;15(2):e0228179. doi: 10.1371/journal.pone.0228179 (PMC7046267; doi:10.1371/journal.pone.0228179)
Supplement: S4 Fig — (PDF) [file pone.0228179.s008.pdf]

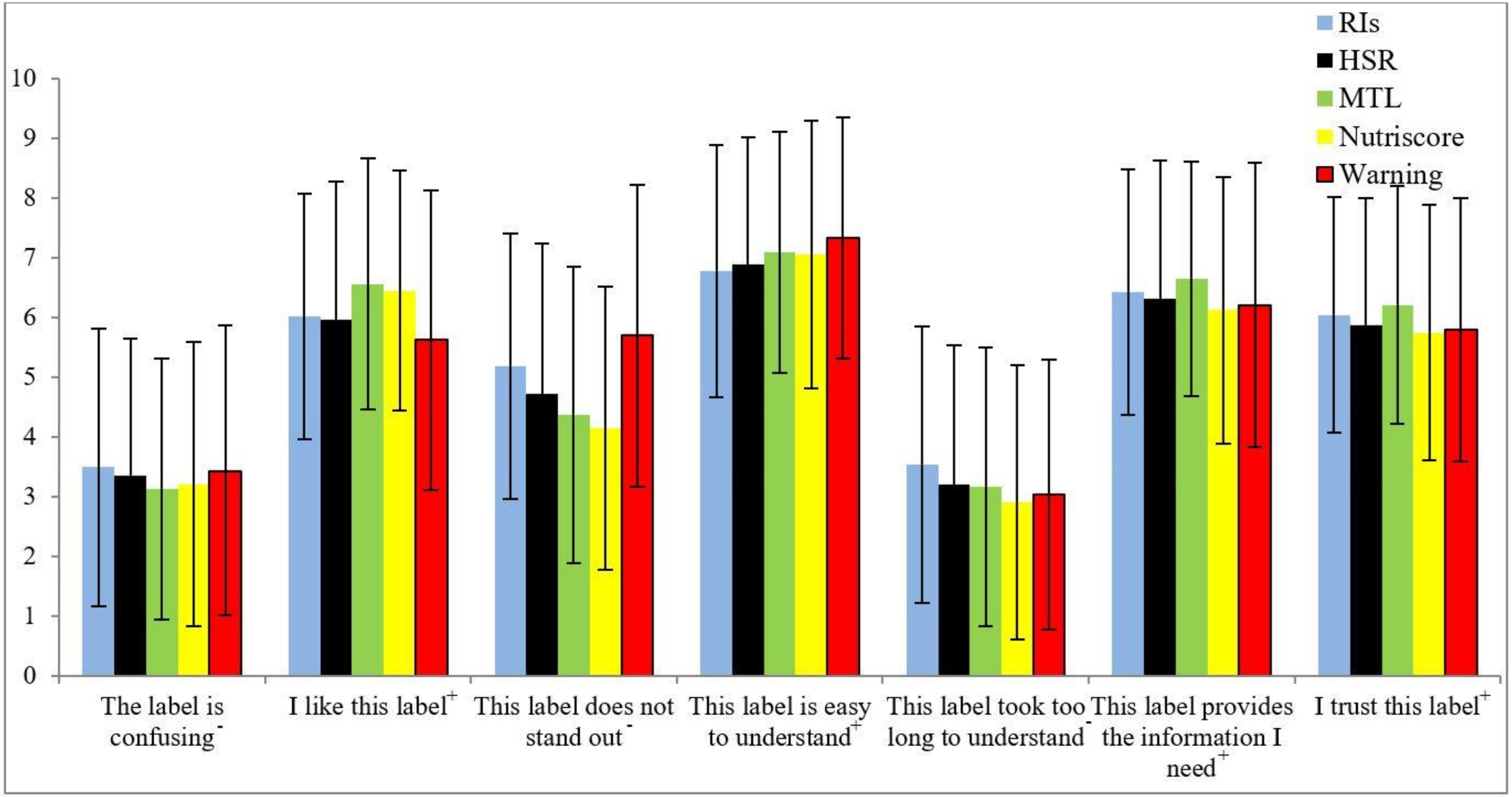

S4 Fig. Average scores for perception questions

+ Positively valanced item; - Negatively valanced item
